# Quality of Life and Psychological Functioning in Children with PFAPA Syndrome

**DOI:** 10.3390/pediatric17030051

**Published:** 2025-04-22

**Authors:** Xosé Ramón García-Soto, Maria Isabel Villanueva-Alameda, Jessica Fernández-Solana, Jerónimo J. González-Bernal, Arancha Bernal-Jiménez, Lara Santos-Martín, Juan García-Mellado, Sara Calvo-Simal, Rodrigo Vélez-Santamaría

**Affiliations:** 1 Clinical Psychology, University Hospital of Burgos, 09006 Burgos, Spain; xgarcia@saludcastillayleon.es (X.R.G.-S.); isabelvillanuevaalameda@gmail.com (M.I.V.-A.); abernalj@saludcastillayleon.es (A.B.-J.); lsantosm@saludcastillayleon.es (L.S.-M.); 2 Department of Health Sciences, University of Burgos, 09001 Burgos, Spain; jejavier@ubu.es (J.J.G.-B.); rvsantamaria@ubu.es (R.V.-S.); 3 Psychiatry Service, University Hospital of Burgos, 09006 Burgos, Spain; jgarciame@saludcastillayleon.es; 4 Research Unit, University Hospital of Burgos, 09006 Burgos, Spain; scalvo@hubu.es

**Keywords:** PFAPA, quality of life, psychological functioning, pediatrics, autoinflammatory diseases, children’s mental health

## Abstract

Background/Objectives: This study analyzes the impact of PFAPA syndrome (periodic fever, aphthous stomatitis, pharyngitis, and adenitis) on health-related quality of life (HRQoL) and the psychological functioning of children and adolescents aged 2 to 1 years. Methods: A cross-sectional descriptive study was conducted with 62 participants (31 males and 31 females) diagnosed with PFAPA. The Strengths and Difficulties Questionnaire (SDQ) and the Family Impact Module scale of Pediatric Quality of Life Inventory (PedsQL) were used to assess psychological functioning and HRQoL, respectively. Results: Participants exhibited predominantly low HRQoL, particularly in physical health and emotional functioning. School functioning was also affected. However, social functioning and family relationships showed more favorable scores. A positive correlation was observed between age and emotional symptoms. Family concern was the most significantly impacted aspect. Conclusions: PFAPA syndrome has a significant impact on the HRQoL of affected children and adolescents, particularly in physical and emotional aspects. A holistic approach is necessary for disease management, considering not only physical symptoms but also psychosocial and academic factors, as well as the impact on the family.

## 1. Introduction

Periodic fever, aphthous stomatitis, pharyngitis, and adenitis (PFAPA) syndrome is an autoinflammatory disease with no known genetic basis, predominantly affecting patients in the first decade of life [1]. It is characterized by recurrent episodes of high fever lasting 3 to 6 days, accompanied by at least one of the following symptoms: pharyngitis, cervical lymphadenopathy, and oral aphthae [2]. PFAPA episodes follow a cyclical pattern, recurring every 3 to 9 weeks, often in a predictable manner. The disease tends to persist for several years, with spontaneous remission typically occurring in early adolescence [3,4]. Regarding symptomatology, fever is the hallmark symptom of PFAPA, with peaks reaching 38.5–41 °C. Between episodes, patients are generally in good health and develop normally. 

Pharyngitis occurs in approximately 90% of cases, often with erythematous or exudative tonsils. Oral aphthae, although not always present, are typically small, shallow, and resolve spontaneously by the end of the febrile episode [5]. Cervical adenitis manifests as painful, usually bilateral, lymphadenopathy.

In addition to these primary symptoms, patients may experience other signs and symptoms during episodes, including general malaise and fatigue, abdominal pain (in about 50% of cases), headache, nausea or vomiting, and arthralgia. It is important to note that, despite the recurrence of episodes, PFAPA does not affect the growth or development of the affected children [6].

Although the exact etiology of PFAPA remains unknown, recent studies suggest a dysregulation of the innate immune response, with abnormal inflammasome activation and increased production of pro-inflammatory cytokines, particularly IL-1β [4,7]. This hypothesis is supported by the favorable response to corticosteroids and, in some cases, IL-1 inhibitors [8,9].

The diagnosis of PFAPA is primarily clinical, based on the modified Marshall criteria [10], which include recurrent febrile episodes with an early onset (<5 years) accompanied by at least one of the characteristic symptoms, in the absence of upper respiratory tract infection and neutropenic cycles [11]. During episodes, an increase in inflammatory markers such as C-reactive protein and erythrocyte sedimentation rate is observed, which normalizes between episodes [12,13].

Recurrent episodes of fever and associated symptoms generate a considerable level of stress and anxiety for both the children and their parents. A recent study found that 32% of mothers of patients with PFAPA experienced moderate or high levels of anxiety. This chronic stress can have long-term effects on the emotional well-being of the entire family [14]. Children with PFAPA syndrome often experience a range of psychological issues that significantly affect their quality of life (QoL). The unpredictability of the febrile episodes can lead to anxiety and feelings of helplessness, as children may become increasingly worried about when the next episode will occur [15,16]. This chronic stress can manifest in various behavioral symptoms, such as clinginess, temper tantrums, or disproportionate sadness, as children struggle to cope with their condition [17].

Social interactions may also be negatively affected, as frequent school absences or lack of opportunities to play due to fever episodes can lead to feelings of isolation and difficulties in maintaining friendships [17]. Additionally, cases of comorbidity between PFAPA and anxiety disorders have been observed, highlighting the potential influence of recurrent febrile episodes on mental health. Emotional stress can also act as a trigger for fever episodes, emphasizing the importance of psychosocial factors in the dynamics of this disease [18,19].

In the last decade, evidence has accumulated on the relationship between self-limiting inflammatory processes and psychopathological alterations, in particular, difficulties in emotional regulation. It is possible, thus, that in addition to the emotional consequences of the interference in daily life caused by the disease, and the vulnerability to stress of patients with PFAPA, there is a direct relationship between the alterations that are at the basis of the disease and emotional difficulties [20,21,22]. It is important to highlight that the impact of recurrent fever due to PFAPA on the QoL of affected children and their families is significant. PFAPA syndrome is a childhood condition that, although not life-threatening, can significantly affect the QoL of both the children and their families. Recent studies have shown that children with PFAPA experience a significantly reduced QoL compared to their healthy peers, with lower scores on health-related quality of life (HRQoL) scales, particularly in the areas of physical and psychosocial functioning [23].

The main objective of this study is to analyze the impact of PFAPA syndrome on children and adolescents aged 2 to 18 years. Specifically, the goal is to assess the patients’ QoL, addressing both their physical and psychosocial well-being. Additionally, this study aims to identify and analyze the presence and intensity of psychopathological symptoms that may be associated with this condition. Finally, this study seeks to explore the parenting challenges faced by the families of these children, in order to better understand the challenges of managing the syndrome and provide useful information for its approach.

## 2. Materials and Methods

### 2.1. Study Design and Participants

This cross-sectional descriptive study was conducted at the University Hospital of Burgos (Burgos, Spain). A sample of 62 children and adolescents (31 males and 31 females), aged between 2 and 18 years, were included in the study. Participants were recruited through an announcement made in the communication network of the PFAPA Syndrome Association of the Island of PFAPA.

Participants were selected based on the following inclusion criteria: a confirmed diagnosis according to the clinical criteria of Thomas, Feder et al. [24], regular follow-up by a healthcare provider with at least one recorded consultation in the past year, and active disease evidenced by recurrent fever episodes reported during the last follow-up visit. The exclusion criteria included minors who declined to participate, tested positive for genetic conditions related to autoinflammatory diseases, or provided invalid contact information, such as incorrect phone numbers or email addresses.

### 2.2. Procedure

Families who decided to participate signed an informed consent form. They were then provided with a link to an application where they completed an online questionnaire (https://forms.office.com/e/b0dyNhvVtz accessed on 1 March 2024) that collected sociodemographic variables of the child, disease-related data, and evaluations of emotional behavior, pediatric quality of life and parenting difficulties. Specifically, parenting difficulties were assessed using the Family Impact Module scale of the Pediatric Quality of Life Inventory (PedsQL) and the prosocial behavior subscale of the Strengths and Difficulties Questionnaire (SDQ). Clinical data from health professionals were not included in the study since the form was distributed online to different participants with PFAPA from different parts of Spain and other countries, which has made it impossible to include these data.

This study was approved by the Bioethics Committee of the University of Burgos (IO 38/2024). Additionally, the research project was submitted to the Medical Research Ethics Committee of the Burgos Health Area and received the required authorization with registration number CEIm 3192. Data were anonymized to ensure patient confidentiality. The data handling complied with the General Data Protection Regulation of the European Union (Regulation EU 2016/679) and the Organic Law 3/2018 on Personal Data Protection and the guarantee of digital rights.

### 2.3. Assessments

Sociodemographic data (gender, age, city of residence, living environment, country of birth) and disease-related variables (onset date of the disease, duration of episodes, maximum body temperature reached, days with fever above 38 °C, number of episodes per year, number of symptom-free days between episodes, and symptoms experienced during episodes) were collected.

Information on the qualities and difficulties of the children and adolescents was gathered using the SDQ, and their quality of life was assessed using the Family Impact Module scale of PedsQL.

The SDQ questionnaire by Goodman [25] is a tool that detects possible cases of mental health and behavioral disorders in children and adolescents aged 2 to 18 years. It exists in several versions to meet the needs of researchers, clinicians, and educationalists. All versions of the SDQ ask about 25 attributes, some positive and others negative. Each item is rated as 0 (not true), 1 (somewhat true), or 2 (certainly true). These 25 items are divided between 5 scales (1) emotional symptoms (5 items), (2) conduct problems (5 items), (3) hyperactivity/inattention (5 items), (4) peer relationship problems (5 items), (5) prosocial behavior (5 items)). Items (1) to (4) are added together to generate a total difficulties score (based on 20 items). The same 25 items are included in questionnaires for completion by the parents of 2–4, 4–17 year olds [25,26]. Questionnaires for self-completion by adolescents ask about the same 25 traits, though the wording is slightly different [26] This self-report version is suitable for young people aged around 11–18.

The SDQ is being used as a research and screening tool throughout the world—in developmental, genetic, social, clinical, and educational studies. It has good psychometric properties and has been translated and validated in Spanish [27,28].

The PedsQL scale [29], is a widely used instrument to assess HRQoL in children and adolescents aged 2 to 18 years. The Family Impact Module scale of PedsQL is a version for completion by parents and versions for self-reporting. It uses a five-point Likert scale to answer questions referring to the past month. The scoring ranges from 0 (never a problem) to 4 (almost always a problem). These scores are linearly transformed into a scale of 0 to 100, with higher scores indicating better HRQoL.

The results obtained in the Family Impact Module scale of PedsQL scale were classified into three categories based on the total mean scores, providing a clear and meaningful interpretation of the patients’ well-being. According to the established classification, a high HRQoL was considered when the mean score was between 81 and 100, indicating excellent HRQoL in all evaluated aspects. A moderate HRQoL was defined for those with a mean score between 60 and 80, reflecting moderate well-being, where patients experience some limitations or difficulties in certain areas of their life. Finally, a low HRQoL was considered when the mean score was below 60, suggesting that patients face significant challenges in multiple aspects, such as health, emotional functioning, or social relationships [30]. The scale shows adequate internal consistency for both self-report and parent versions. Its construct validity is appropriate, and it has proven to be sensitive to changes in the health status of children. It has been validated in Spain [31,32,33].

### 2.4. Statistical Analysis

Descriptive analyses were conducted on the sociodemographic and clinical characteristics of the sample, expressing categorical variables in absolute frequencies and percentages, and continuous variables in means and standard deviations (SD). The normality of the dataset was assessed using the Kolmogorov–Smirnov test.

To evaluate the association between gender, emotional behavior, and QoL, the Student’s *t*-test for independent samples was used. The relationship between age and these same variables was analyzed using Pearson’s correlation.

The statistical analysis was performed using SPSS version 28 (IBM-Inc., Chicago, IL, USA). Statistical significance was determined with a *p*-value < 0.05.

## 3. Results

The sample of this study consisted of 62 children and adolescents, with an equal gender distribution (31 males and 31 females), aged between 2 and 18 years (mean age of 6.85 ± 2.96 years). Participants came from various countries, primarily Spain (*n* = 55), Argentina (*n* = 3), Mexico (*n* = 2), Guatemala (*n* = 1), and Uruguay (*n* = 1).

Regarding the age of onset of the syndrome, the mean age was 1.66 years, with a significant majority of cases diagnosed before the age of three (93.5%), highlighting the early onset of the disease in the studied population.

Regarding the most common symptoms during PFAPA episodes, it was observed that pharyngitis (96.8%), cervical lymphadenopathy (90.3%), and aphthous stomatitis (74.2%) were the most prevalent, consistent with the typical symptoms described in the literature for this syndrome. Additionally, a considerable percentage of children also experienced headaches (75.8%) and abdominal pain (80.6%), symptoms that may influence the quality of life (QoL) of the children and their families.

The average duration of episodes was 5.58 ± 4.03 days, with a fever lasting an average of 2.85 ± 1.87 days and reaching a maximum temperature of 40.43 °C ± 0.89. This pattern of recurrent symptoms resulted in an average of 12.22 ± 6.98 episodes per year, with significant variability (ranging from 2 to 30 episodes), emphasizing the unpredictable and chronic nature of the disease. Despite the frequency and severity of the episodes, patients had an average symptom-free period of 19.64 ± 11.58 days between episodes, suggesting that although the episodes are recurrent, there is notable variability in the time between them.

### 3.1. Qualities and Difficulties in Children and Adolescents with PFAPA

In the following Table 1, the results obtained in the different dimensions of the SDQ are presented, offering an overview of the emotional and behavioral aspects of the participants.

In terms of emotional symptoms, the average score suggests that participants exhibit moderate levels of emotional problems. Regarding behavioral problems, a low frequency of disruptive behaviors was reported among the participants. In hyperactivity, the average reflected low levels in the sample. In the dimension of peer problems, it was observed that difficulties in relationships with peers were minimal among the participants. Finally, in the prosocial behavior dimension, where the highest average score was obtained, participants exhibited positive prosocial behavior, characterized by empathy, cooperation, and interpersonal skills.

No statistically significant differences were found between males and females in relation to symptoms across the different SDQ dimensions. However, a significant difference was observed in the prosocial behavior dimension, with higher scores for females (*p* = 0.018) compared to males. Despite this difference, both genders were within the satisfactory scoring range, with females obtaining a mean of 8.419 ± 1.607 and males a mean of 7.354 ± 1.836.

Additionally, no statistically significant relationships were found in the SDQ dimensions based on the participants’ age, except for the emotional symptom dimension, where a statistically significant positive correlation was obtained (*p* < 0.001), indicating that as the participants’ age increased, their emotional symptoms also increased (Table 2).

### 3.2. Health-Related Quality of Life in PFAPA

The results obtained for each aspect evaluated through the PedsQL questionnaire are shown in Table 3. In general terms, the results revealed that the participants had predominantly low HRQoL in several of the evaluated aspects. Health was one of the most critical aspects, with a mean of 58.85.

Emotional functioning also showed low scores, with a mean of 57.27. In contrast, social functioning had a mean score of 67.03. The dimension of school activities was also affected, with a mean of 59.04, suggesting that participants face significant obstacles in their school performance due to their medical condition.

The aspect related to communication obtained a mean score of 60.81. Regarding cognitive functioning, the mean was 72.05, placing it in the category of medium HRQoL, indicating that, in general, the patients did not show significant cognitive deficiencies, though there was moderate variability in the scores.

Regarding family relationships, the mean score was 73.45, suggesting that, in general, patients reported satisfactory family relationships. However, the most affected aspect was related to family concerns, which obtained the lowest score, with a mean of 33.75. This highlights the significant worry experienced by the families of children with PFAPA.

No significant statistical differences were found between males and females regarding the scores obtained on the PedsQL scale. On the other hand, the correlation between different areas or variables was analyzed using Pearson’s correlation coefficient (Table 4). A statistically significant correlation was observed between age and school activities (r = 0.422, *p* = 0.003), indicating that as children grow, they tend to face greater challenges in the school setting. This finding may reflect the increasing complexity of academic demands as children and adolescents develop.

Similarly, the correlation between family relationships and social functioning (r = 0.472, *p* = 0.007) shows that better family relationships are associated with better social functioning. The correlation between physical functioning and family concerns (r = 0.591, *p* = 0.043) shows that the greater the perceived physical difficulties, the greater the family concern. This finding could suggest that parents of children with greater physical limitations tend to experience more worries related to their child’s health and well-being.

A significant positive correlation was observed between physical functioning and cognitive functioning (r = 0.496, *p* = 0.022). This result suggests that children with better physical functioning also tend to have better cognitive functioning. The correlation between physical functioning and emotional functioning (r = 0.626, *p* = 0.022) indicates a moderately strong relationship between these two dimensions. Finally, a significant correlation was found between school activities and communication (r = 0.659, *p* < 0.001). This result suggests a strong positive relationship, indicating that children who experience greater difficulties in school activities also tend to have challenges in their communication abilities.

## 4. Discussion

The objective of this study was to analyze the impact of PFAPA syndrome in children and adolescents aged 2 to 18 years, evaluating their HRQoL and the presence of psychopathological symptoms. Additionally, this study aimed to explore the parenting difficulties faced by the families of these minors.

The studied sample presented an equitable gender distribution and a mean age of 6.85 years, with an early onset of the disease. This highlights the pediatric nature of PFAPA and the importance of early diagnosis and management. These findings are consistent with previous studies that reported an average age of onset of 1.58 years in a cohort of Romanian children with PFAPA [34].

The observed symptoms, with a high prevalence of pharyngitis, cervical lymphadenopathy, and aphthous stomatitis, are consistent with the diagnostic criteria established for PFAPA [19]. Additionally, a high percentage of children with headache and abdominal pain was observed, which could be related to emotional comorbidities such as anxiety, a factor that could also influence the quality of life of the children and their families. These symptoms have also been reported in other studies, such as Grimwood et al. [23], who found a high prevalence of fatigue in children with PFAPA.

The results obtained reflect the recurrent and unpredictable nature of PFAPA syndrome, which can have a significant impact on the quality of life of the patients. The variability observed in the frequency and duration of episodes suggests that the disease does not follow a uniform pattern, complicating its clinical management and the prediction of its progression. This variability was also observed by Sparud-Lundin et al. [19] who highlighted how the unpredictability of the episodes significantly affects the daily lives of families.

Furthermore, our results suggest that although children and adolescents with PFAPA may experience moderate levels of emotional problems, behavioral disturbances are low. The low frequency of disruptive behaviors and the low levels of hyperactivity suggest that the disease does not appear to be associated with significant behavioral alterations. Additionally, difficulties in peer relationships were minimal, suggesting that PFAPA does not have a significant impact on the social interactions of the participants. These findings partially contrast with another study [23], who found a significant impact on the psychosocial functioning of children with PFAPA, especially in the preschool age group.

However, it is important to note that other studies have found results similar to ours. For example, Karayağmurlu et al. [35] observed significant improvements in the emotional and behavioral functioning of children with PFAPA after surgical intervention, suggesting that behavioral problems, when present, can be manageable. Additionally, another study found that although PFAPA significantly affects family life, children generally maintain adequate social functioning between febrile episodes [19].

It was noted that there were no statistically significant differences between boys and girls in most of the SDQ dimensions, except in prosocial behavior, where girls had significantly higher scores than boys. Despite this difference, both genders showed satisfactory levels in this dimension, suggesting that, regardless of gender, participants demonstrated positive behaviors such as empathy and cooperation. This finding is consistent with previous studies that have found that girls tend to score higher in prosocial behaviors compared to boys. For example, one study found that girls exhibited higher levels of empathy and cooperation in various social situations, reinforcing the idea that gender differences may influence prosocial behavior from an early age [36].

No significant relationships were found between age and the SDQ dimensions, except for emotional symptoms, which showed a positive correlation. This suggests that older participants tend to exhibit higher levels of emotional problems. Given that PFAPA is generally considered a benign, self-limiting condition that remits by adolescence, the increase in emotional symptoms when the disease, at least in theory, is in remission appears counterintuitive. One possible explanation is that the emotional symptoms reflect underlying alterations linked to the inflammatory process itself. Recent literature [20,21,22] highlights the potential role of neurobiological mechanisms in autoimmune inflammation, which may disrupt emotional regulation. Thus, recurrent PFAPA episodes could contribute to emotional dysregulation through broader neuroimmunological pathways rather than disease-specific symptomatology. Recurrent systemic inflammation in PFAPA activates key neuroimmunological pathways linking proinflammatory cytokine dysregulation with alterations in emotional regulation. Recent studies demonstrate that IL-1β, central to the pathophysiopathology of PFAPA [37,38], crosses the blood–brain barrier via active transport, inducing microglial activation and altering synaptic plasticity in limbic regions [39,40]. This process is enhanced by IL-6, whose elevated levels during episodes reduce hippocampal neurogenesis and increase amygdala reactivity, correlating with the internalizing symptoms observed in adolescents [36]. IL-1β activates indo-leamine 2,3-dioxygenase (IDO), diverting tryptophan metabolism towards the production of quinurenic acid (neurotoxic) instead of serotonin. This could explain the positive correlation between age and emotional symptoms, given the cumulative effect of recurrent episodes [37,40]. The results obtained through the PedsQL questionnaire indicate that the HRQoL of the participants is predominantly low in several dimensions, with physical health and emotional functioning being the most affected aspects. Likewise, this aspect may also be influenced by the neurobiological aspect of autoimmune inflammation referring to chronic exposure to IFN-γ (elevated in PFA-PA) that reduces amygdala-dorsolateral prefrontal cortex functional connectivity, a pattern observed in anxiety disorders. This is reflected in our study by the impairment in emotional functioning shown by PedsQL participants [39,40].

These findings are consistent with recent scientific literature; a study demonstrated that the HRQoL of children with PFAPA is significantly lower compared to their healthy peers, particularly in the physical health and psychosocial functioning dimensions [23]. This finding supports our observations of low HRQoL in various dimensions, particularly in physical health and emotional functioning.

Regarding school functioning, this is also compromised, suggesting that the illness may interfere with school performance. Our findings regarding this compromise are supported by a study in which children with PFAPA were found to experience significant sleep problems, which could affect their school functioning [41]. The authors demonstrated a positive correlation between the duration of the illness and sleep problems, suggesting an accumulative impact on daily functioning, including school functioning. In contrast, social functioning and family relationships showed more favorable scores, indicating that despite the difficulties, children and adolescents with PFAPA may maintain satisfactory social and family interactions. This finding contrasts with the results of a qualitative study that revealed that parents of children with PFAPA experience increased stress, constant fatigue, and restrictions in family social life [19].

The correlation analysis revealed that as children grow, they face greater challenges in the academic field, possibly due to the increasing academic complexity. It was also found that better quality of family relationships is associated with higher social functioning, while an increase in perceived physical difficulties leads to greater concern among families. Additionally, it was evident that better physical functioning is related to better cognitive and emotional functioning, highlighting the interdependence between various dimensions of well-being.

Finally, family concern emerged as the most affected aspect, suggesting that the emotional burden for caregivers is significant and should be considered in the comprehensive management of the disease. Our work shows unmet needs of PFAPA patients and their families, in particular, aspects related to physical functioning, school activity, and emotional functioning of patients. These results show that it is necessary to improve our knowledge of the daily functioning of patients and their families in order to design support and intervention strategies to address their psychosocial needs. These results are in line with those of other research [42]. As suggested in several studies, the mental and physical burden of the family is fundamental and should be part of comprehensive approaches and work not only with the child or adolescent with PFAPA but also with the family. It was determined that the use of special pedagogical and psychological approaches can be effective for this purpose. As highlighted in other studies, the psychological and physical burden experienced by families is a critical factor in PFAPA management. Comprehensive care should, therefore, extend beyond the affected child or adolescent to include family-centered support. Evidence suggests that tailored pedagogical and psychological interventions can be highly effective in this context. Such a multidisciplinary approach can help alleviate the disease’s impact on both familial and school environments [43,44]. Some qualitative studies reveal that families may need structured crisis management education that includes school protocols, as well as psychological interventions to coexist with chronic stress (family therapy, relaxation techniques, or parenting support groups) and institutional supports such as personalized educational plans with flexibility in attendance and academic assessment [18,45,46]. These tools allow for customized educational support through accommodations such as breaks during classes, access to food and water, and tutoring to make up for missed content. In addition, training programs for teachers on autoinflammatory diseases can improve school integration and reduce the stigma associated with recurrent absences [47]. The exploration of HRQoL in children with PFAPA syndrome provides important insights into the impact of the disease on patients and their families. The initial findings from pilot studies indicate that the HRQoL of patients with PFAPA is considerably affected, challenging the previous notion that PFAPA is a benign disease. Further research is needed to expand these findings and deepen our understanding of the disease.

To validate the preliminary results of the current studies, larger multi-center studies are essential. These studies should aim to include a broader range of patients to better correlate HRQoL with validated scores of disease severity and activity. The novelty and limitation of available data may explain why, in the pediatric and rheumatological literature of our environment [48], references to potential relationships between PFAPA and quality of life or psychopathological difficulties are scarce. This situation favors a healthcare focus limited to the medical aspects of episodes, without identifying the possible psychological difficulties of children and their families, leaving their situation unaddressed.

Finally, we must highlight some limitations of this study. First, its cross-sectional design does not allow us to capture changes in quality of life over time, which is particularly relevant given the fluctuating course of PFAPA. Symptoms can vary significantly across different periods, meaning that some findings may not reflect the overall patient experience but only their condition at the time of data collection. Therefore, future research should adopt a longitudinal approach to gain a deeper understanding of the long-term effects of PFAPA on children’s psychological well-being and quality of life, as well as the effects of the different modalities of treatment of the disease. PFAPA is sometimes treated with corticosteroids. In other cases, surgical removal of the tonsils is performed, which may lead to improvement of symptoms. More generally, various treatments are used throughout the course of the disease. This raises a question as to whether different forms of treatment and therapy have varying effects on the psychological well-being and quality of life of children [9,49].

Other potential limitations relate to the psychometric instruments used in our study. On one hand, the scales used to assess psychopathological symptoms and HRQoL explored difficulties present in the month prior to the administration of the scale. However, as we have seen, the number of annual episodes among our subjects varies widely, ranging from 2 to 30 episodes. This may have led to an underrepresentation of the difficulties experienced by subjects who had been asymptomatic for more than a month at the time of assessment. If this interpretation is confirmed, it is possible that our results underestimate the problems faced by children with PFAPA syndrome and their families. On the other hand, although this study used validated tools (SDQ and PedsQL), these measures may be influenced by emotional or contextual factors. Nevertheless, we consider that the results provide valuable preliminary insights into the family and personal circumstances of children with PFAPA syndrome—the primary objective of this study. Future studies should incorporate objective measures (e.g., clinical records documenting episode frequency and severity) to complement these findings. Such an approach would enable a more comprehensive evaluation of PFAPA’s true impact and its association with family perceptions. Additionally, including a control group would address methodological limitations inherent to studies relying solely on psychometric assessments.

It is also necessary to mention the impossibility of accessing the medical data of the participants, given that, being a little-known disease, it has been necessary to collect data through online forms from participants from all over Spain and other countries. Finally, although key needs such as emotional support were identified, this study did not evaluate specific family support interventions. Future research should quantify the impact of structured programs that combine multidisciplinary coordination and training in emotional regulation techniques, as well as, for example, digital platforms for symptomatic monitoring and early warning.

## 5. Conclusions

The results of this study showed that the emotional and behavioral aspects of the participants showed low levels of hyperactivity, with a low frequency of conduct problems and difficulty in relating with peers, as well as positive prosocial behavior with the presence of empathy, cooperation, and interpersonal skills. However, they presented moderate levels of emotional symptomatology. Significant differences were found in prosocial behavior between men and women, with women showing higher levels, although both sexes presented satisfactory ranges. Also noteworthy is a positive correlation between the emotional symptomatology of the participants and age. Given that, as PFAPA is currently identified, it is a benign condition that remits spontaneously from adolescence onwards, it is surprising that the emotional symptoms become evident when the disease, at least in theory, is remitting. One possible interpretation is that the emotional symptoms are another manifestation of the alterations that, in turn, underlie the inflammatory process, which could suggest the presence of mechanisms underlying these effects, such as neurobiological aspects of autoimmune inflammation.

Also, in the results of this study, it was possible to observe that participants had a predominantly low HRQoL in several dimensions, particularly in physical health and emotional functioning. School functioning was also affected, suggesting that the disease interferes with school performance. However, social functioning and family relationships showed more favorable scores, indicating that children with PFAPA can maintain satisfactory social and family interactions despite the challenges.

The correlation analysis revealed that academic challenges increase with age, possibly due to growing academic complexity. It was found that better family relationship quality is associated with higher social functioning, while perceived physical difficulties lead to greater family concern. This study also highlighted an interdependence between physical, cognitive, and emotional functioning.

Family concern emerged as the most affected aspect, suggesting a significant emotional burden for caregivers that should be considered in the comprehensive management of the disease. Therefore, it is suggested that they participate in comprehensive approaches through special pedagogical and psychological approaches involving the person affected with PFAPA and their family members, such as family therapy interventions, support groups for parents, or personalized educational support in order to mitigate the impact of the disease on the school and family environment.

## Figures and Tables

**Table 1:** Results of the Strengths and Difficulties Questionnaire (SDQ).

	Media	SD
Emotional Symptoms	5.112	2.548
Behavioral Problems	2.467	2.022
Hyperactivity	2.403	2.130
Peer Problems	2.112	2.000
Prosocial Behavior	7.887	1.793

**Table 2:** Pearson correlation coefficient between the different dimensions of the Strengths and Difficulties Questionnaire (SDQ).

		Age	Total Scale	Emotional Symptoms	Behavioral Problems	Hyperactivity	Peer Problems	Prosocial Behavior
Age	Pearson Correlation	1	0.026	0.396 **	−0.216	−0.145	−0.055	0.164
	Sig. (two-tailed)		0.840	<0.001	0.092	0.260	0.670	0.204
	N		62	62	62	62	62	62

** *p* < 0.001.

**Table 3:** Scores obtained on the Pediatric Quality of Life Scale (PedsQL).

	Mean	SD
Health	58.854	21.150
Emotional Functioning	57.272	25.435
Social Functioning	67.031	23.427
School Activities	59.042	20.176
Communication	60.810	27.206
Cognitive Functioning	72.045	17.331
Family Relationships	73.452	19.015
Family Concerns	33.750	26.639

**Table 4:** Pearson’s correlation coefficient between age and the different dimensions of the PedsQL in children and adolescents with PFAPA syndrome.

		Health	Family Concerns	School Activities	Family Relationships	Communication	Cognitive Functioning	Social Functioning	Emotional Functioning
Age	Pearson Correlation	−0.231	−0.053	0.422	0.243	−0.033	0.169	0.260	0.300
	Sig. (two-tailed)	0.277	0.773	0.003	0.121	0.844	0.273	0.105	0.175
	N	24	32	47	42	37	44	40	22

## Data Availability

Data for this research are available upon request from the corresponding author.

## Abbreviations

The following abbreviations are used in this manuscript:

HRQoL	Health-Related Quality of Life
PFAPA	Periodic Fever, Aphthous Stomatitis, Pharyngitis, and Adenitis Syndrome
PedsQL	Pediatric Quality of Life Scale
QoL	Quality of Life
SD	Standard Deviation
SDQ	Strengths and Difficulties Questionnaire
